# Genome‐wide comparisons reveal a clinal species pattern within a holobenthic octopod—the Australian Southern blue‐ringed octopus, *Hapalochlaena maculosa* (Cephalopoda: Octopodidae)

**DOI:** 10.1002/ece3.3845

**Published:** 2018-01-25

**Authors:** Peter Morse, Shannon R. Kjeldsen, Mark G. Meekan, Mark I. Mccormick, Julian K. Finn, Christine L. Huffard, Kyall R. Zenger

**Affiliations:** ^1^ Australian Institute of Marine Science UWA Oceans Institute Crawley WA Australia; ^2^ College of Science and Engineering James Cook University Townsville Qld Australia; ^3^ Museums Victoria Melbourne Vic. Australia; ^4^ Monterey Bay Aquarium Research Institute Moss Landing CA USA; ^5^ California Academy of Sciences San Francisco CA USA

**Keywords:** adaptive radiation, cryptic subspecies, ecological genomics, population genetics, SNP

## Abstract

The southern blue‐ringed octopus, *Hapalochlaena maculosa* (Hoyle, 1883) lacks a planktonic dispersal phase, yet ranges across Australia's southern coastline. This species’ brief and holobenthic life history suggests gene flow might be limited, leaving distant populations prone to strong genetic divergence. This study used 17,523 genome‐wide SNP loci to investigate genetic structuring and local adaptation patterns of *H. maculosa* among eight sampling sites along its reported range. Within sites, interrelatedness was very high, consistent with the limited dispersal of this taxon. However, inbreeding coefficients were proportionally lower among sites where substructuring was not detected, suggesting *H. maculosa* might possess a mechanism for inbreeding avoidance. Genetic divergence was extremely high among all sites, with the greatest divergence observed between both ends of the distribution, Fremantle, WA, and Stanley, TAS. Genetic distances closely followed an isolation by geographic distance pattern. Outlier analyses revealed distinct selection signatures at all sites, with the strongest divergence reported between Fremantle and the other Western Australian sites. Phylogenetic reconstructions using the described sister taxon *H. fasciata* (Hoyle, 1886) further supported that the genetic divergence between distal H. maculosa sites in this study was equivalent to that of between established heterospecifics within this genus. However, it is advocated that taxonomic delineations within this species should be made with caution. These data indicate that *H. maculosa* forms a clinal species pattern across its geographic range, with gene flow present through allele sharing between adjacent populations. Morphological investigations are recommended for a robust resolution of the taxonomic identity and ecotype boundaries of this species.

## 1 INTRODUCTION

Dispersal is an important component of animal life histories that influences habitat expansion and the maintenance of population connectivity along the geographic ranges of species (Barton, 1992). Most marine invertebrates and fish species have a biphasic life history, with a pelagic larval stage that allows them to take advantage of ocean currents for dispersal from natal sites (Gilg & Hilbish, 2003). This phase enables these organisms to find suitable habitats for settlement and minimizes an individual's competition with conspecifics for resources at localized sites (Caley et al., 1996). Furthermore, efficient dispersal mechanisms result in greater genetic connectivity among populations, and this reduces the possibility of inbreeding depression (Charlesworth & Charlesworth, 1987; Gilg & Hilbish, 2003).

Previous molecular studies of the Cephalopoda have revealed that genetic structuring of populations generally mirrors life history traits (Cabranes, Fernandez‐Rueda, & Martínez, 2008; Higgins, Semmens, Doubleday, & Burridge, 2013; Kassahn et al., 2003; Semmens et al., 2007; Shaw, Pierce, & Boyle, 1999). For example, the squids (Cephalopoda: Teuthida), all of which have planktonic larvae and are nektonic in their adult stage (Boletzky, 1987), are commonly reported to have high levels of gene flow over large spatial scales (Carvalho, Thompson, & Stoner, 1992; Garthwaite, Berg, & Harrigan, 1989; Reichow & Smith, 2001; Shaw et al., 1999). Ecologically relevant differentiation among populations in squid taxa has only been observed over very large distances (ocean basins) or in the presence of a geographic barrier to dispersal (Carvalho et al., 1992; Garthwaite et al., 1989; Shaw et al., 1999). Contrastingly, genetic studies of cuttlefish (Cephalopoda: Sepiidae), which have no planktonic phase (Boletzky, 1987), consistently show genetic structuring at relatively fine scales across species ranges (Kassahn et al., 2003; Pérez‐Losada, Guerra, Carvalho, Sanjuan, & Shaw, 2002; Zheng et al., 2009). Population structuring in cuttlefish typically follows an “isolation by distance” (IBD) pattern (Kassahn et al., 2003; Pérez‐Losada et al., 2002; Wright, 1943) that reflects the sedentary nature of cuttlefish hatchlings (Boletzky, 1987). Following this pattern, proximal populations within a species might be closely related, but the genetic divergence among populations increases proportionally with the geographic distance between them (Wright, 1943).

Adult incirrate octopuses (Octopoda: Incirrina) are the most sedentary of the cephalopods (Cigliano, 1993; Hanlon & Messenger, 1998). Where studied, their population structure greatly depends on whether the species has a holobenthic or merobenthic life cycle (Cabranes et al., 2008; Higgins et al., 2013; Juárez, Rosas, & Arena, 2010). For example, a recent study of two sympatric octopuses, one with a planktonic larval phase (merobenthic) and the other without (holobenthic), suggested that this life history trait may drive the type of genetic structuring among populations of these species (Higgins et al., 2013). In the case of the former species, the merobenthic Maori octopus (*Macroctopus maorum* Hutton, 1880), population connectivity was predominantly influenced by ocean currents (Doubleday, Semmens, Smolenski, & Shaw, 2009; Higgins et al., 2013). Contrastingly, genetic structure of the holobenthic pale octopus (*Octopus pallidus* Hoyle, 1885) followed an IBD pattern common to cuttlefish and many terrestrial animals (Higgins et al., 2013; Kassahn et al., 2003; Pérez‐Losada et al., 2002; Wright, 1943).

The above studies are useful for advancing hypotheses about the dispersal processes leading to population structure among cephalopod taxa. However, to our knowledge, there have been no studies addressing the broad‐scale patterns of genomic differentiation or adaptive radiation of a holobenthic cephalopod along its entire species range. Theory would suggest that reduced gene flow would leave populations of holobenthic cephalopods particularly susceptible to genetic divergence due to both increased random drift and differences in selective pressures occurring over varying habitat types (Lenormand, 2002; Mayr, 1963). Such divergence between conspecific populations based on local adaptation over time can lead to the evolution of cryptic subspecies and/or speciation (Doebeli & Dieckmann, 2003; Kirkpatrick & Barton, 2006).

**Figure 1:**
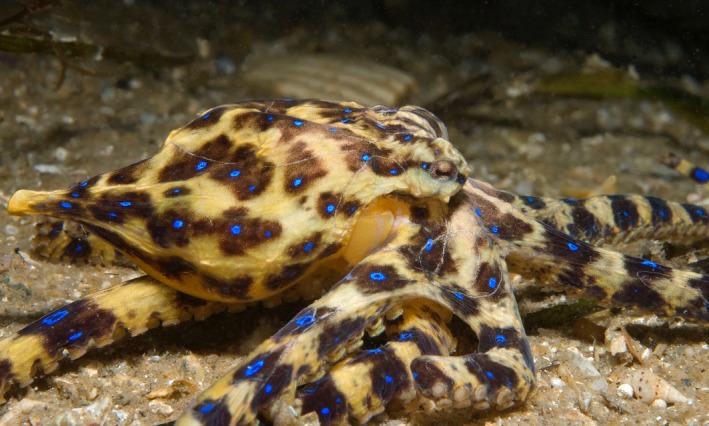
An image is shown of the southern blue‐ringed octopus (*Hapalochlaena maculosa*) from Port Phillip Bay, Victoria (Photo taken by Julian Finn, Museums Victoria)

The southern blue‐ringed octopus (*Hapalochlaena maculosa*; Figure 1) provides a unique model for addressing biological questions related to mechanisms of population divergence and gene flow. This is due to many unique aspects of this species’ distinctive life history. *H. maculosa* is holobenthic and has a brief 7‐month life cycle that terminates in a single breeding season (Tranter & Augustine, 1973). Fecundity in this species is relatively low compared to other cephalopod taxa (Boyle, 1987; Tranter & Augustine, 1973), with females producing up to approximately fifty eggs (Tranter & Augustine, 1973). The mothers invest heavily into their egg clutch by cleaning and guarding the eggs over a 2‐month embryonic development phase, until the time of hatching and the mother's eventual senescence and death (Tranter & Augustine, 1973). This extended embryonic phase and maternal care leads to direct development of the offspring (Tranter & Augustine, 1973). Upon hatching, juvenile *H. maculosa* are immediately confined to the benthic environment (Tranter & Augustine, 1973). Juveniles attain sexual maturity after approximately 4 months of growth, after which they spend most of their time seeking out mates (Tranter & Augustine, 1973).

Throughout its life cycle, *H. maculosa* is capable of swimming only very short distances, via jet propulsion from the siphon (Tranter & Augustine, 1973). Despite its presumably limited dispersal capacity due to the lack of a planktonic phase, *H. maculosa* is widespread along the entire Southern Ocean coastline of the Australian continent (Jereb, Roper, Norman, & Finn, 2014). Additionally, on the subtropical west coast of Australia, an undescribed sister species has been reported, the western blue‐ringed octopus (“WBRO”; Norman, 2000). This potential sister taxon (referred to hereafter as “ecotype”) appears similar to *H. maculosa* in its external morphology and holobenthic life history, but has been delineated based on its possession of a functional ink sac (Norman, 2000). However, the geographic boundary between these distinct ecotypes remains unclear due to a lack of genetic and morphological data along this part of the genus range, and both ecotypes will be considered as part of the “*H. maculosa* group” here for simplicity.

It is hypothesized that *H. maculosa* and the WBRO might interbreed at population boundaries and that limited gene flow between all adjacent populations might lead to a clinal species pattern (see Slatkin, 1973) along the southwestern and southern coasts of Australia. This could potentially result in a gradient‐like species complex, until the range reaches the described species distribution of the blue‐lined octopus (*H. fasciata*) on the subtropical eastern coast, or environments become too warm on the tropical west coast (Jereb et al., 2014). It is also hypothesized that the inferred limited dispersal of these animals, combined with differences in selective pressures along this taxon's range, such as temperature gradients, depth profiles, or predation risks, could lead to the presence of additionally unique genetic groups and/or possible subspecies within the *H. maculosa* group. Due to their cryptic nature, there is currently very little known about the behavioral ecology or mating system of *Hapalochlaena* spp. that occur along this range (c.f. Morse, Zenger, McCormick, Meekan, & Huffard, 2015, 2017). However, these life history characteristics also have the potential to influence the genetic structure and/or reinforce geographic boundaries between potential subspecies within this group (Wright, 1940).

This study used genome‐wide single‐nucleotide polymorphism (SNP) markers to explore the microevolutionary processes shaping the genetic structure of the *H. maculosa* group across its range. In particular, the genetic diversity and connectivity were compared among eight sample sites along the *H. maculosa* group distribution, from Fremantle, WA, to Stanley, Tasmania. Additionally, genetic signatures of selection were identified at each sampled location in order to estimate the role(s) of local adaptation in driving of the observed genetic divergence between regions. Finally, this study aimed to resolve the phylogenetic relationships among members of the *H. maculosa* group across their geographic distribution and to provide insight for the taxonomic identity of the species group.

## 2 METHODS

### 2.1 Sample collection

**Figure 2:**
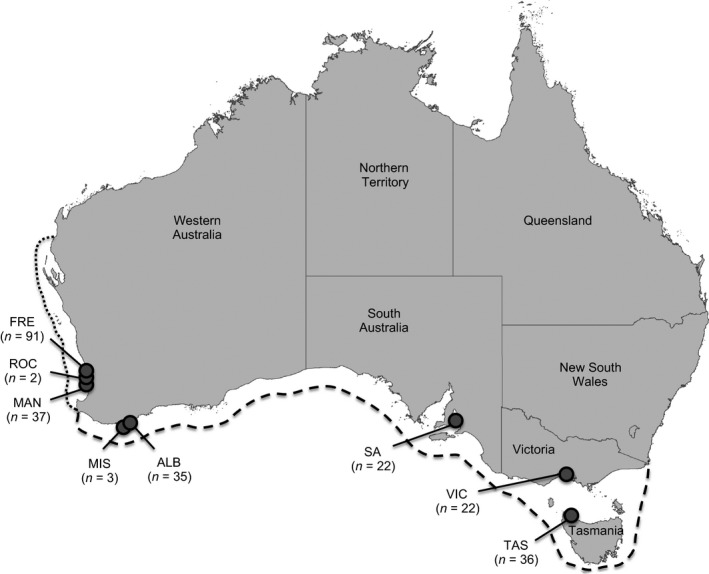
Sampling locations for the 248 members of the *H. maculosa* group sourced in this study. Site names and sample sizes are given next to each location. The reported distribution of *H. maculosa* is shown within the dashed line (Jereb et al., 2014). The subtropical region of Western Australia, previously proposed as the distribution for the undescribed WBRO, is represented with the dotted line (Norman, 2000)

A total of 248 samples from the *H. maculosa* group were sourced from eight sampling sites across the southwestern and southern coastlines of Australia (Figure 2): Fremantle, WA (FRE, *n* = 91; sampling area ≈ 61 km^2^); Rockingham, WA (ROC, *n* = 2; sampling area ≈ 0.1 km^2^); Mandurah, WA (MAN, *n* = 37; sampling area ≈ 220 km^2^); Misery Beach, WA (MIS, *n* = 3; sampling area ≈ 0.1 km^2^); Emu Point (Albany), WA (ALB, *n* = 35; sampling area ≈ 1 km^2^); Gulf St. Vincent, SA (SA, *n* = 22; sampling area ≈ 0.02 km^2^); Port Phillip Bay, VIC (VIC, *n* = 22; sampling area ≈ 0.02 km^2^); and Stanley, TAS (TAS, *n* = 36; sampling area ≈ 22 km^2^). Specimens from the Fremantle, Mandurah, Emu Point, and Stanley sites were obtained through the bycatch of commercial fishermen. Samples from the Rockingham and Misery Beach sites were obtained through false‐shelter traps comprised of both 200 mm lengths of 20‐mm‐diameter PVC pipes and concrete cavity traps (modified from Schafer, 2001) with cavity sizes of 50 × 30 mm. Samples from the Gulf St. Vincent and Port Phillip Bay sites, as well as two *H. fasciata* samples used as a known sister taxon for phylogenetic analyses, were obtained during field surveys by J. Finn. Distal 2‐mm arm segments were sampled from all animals and placed in 70% ethanol until DNA extraction. Due to small sample sizes in the Rockingham and Misery Beach sites, these were only included in phylogenetic analyses and were omitted from all other genetic evaluations. The use and treatment of the animals were approved by the James Cook University Animal Ethics Committee (Approval Number: A1850). Animals were sourced under Western Australia DPaW permit: SF00963, Western Australia Fisheries exemption: 2393, and the Department of Environment and Primary Industries fisheries research permit: RP699.

### 2.2 DNA extraction and genotype by sequencing

DNA was extracted from all tissue samples using a modified CTAB/Chloroform—Isoamyl method (Adamkewicz & Harasewych, 1996) and further purified using Sephadex™ G‐50 spin columns to ensure removal of any small molecule contaminants prior to sequencing (as per Lal, Southgate, Jerry, & Zenger, 2016; Lal, Southgate, Jerry, Bosserelle, & Zenger, 2017). Quality of DNA and visual indicators of contaminants were resolved using a 0.8% agarose gel. All samples were quantified and standardized to a 50 ng/μl concentration using Biotium ACCUBLUE™ High Sensitivity dsDNA quantification kit. Finally, all samples were sent to the genotyping service provider, Diversity Arrays Technology PL, Canberra ACT, Australia, for full restriction enzyme digestion, library preparation, genotype‐by‐sequencing data generation, and QA/QC of sequences via DArTseq™ 1.0 technology (Kilian et al., 2012; Sansaloni et al., 2010). DArTseq 1.0 technology generates two independent genetic marker types—SNPs and presence–absence variant (PAV, dominant loci) markers—identified from restriction site‐associated (RAD) fragments recovered in the sequence data. SNPs were used for both population and phylogenetic analyses, whereas PAVs were only used in phylogenetic reconstructions. Sequence quality control, marker filtering, and genotype calling at Diversity for both marker types are described in Lal et al. (2016, 2017).

### 2.3 SNP and PAV quality control

A total of 33,230 high‐quality unique SNPs (single SNP per sequence tag) and 39,033 unique PAV loci were resolved by DArTseq™. SNPs were filtered for call rate (>70%) and minor allele frequency (MAF; <5% in all six sites with *n* > 20) to ensure high‐quality data. Additionally, all SNP loci deviating from Hardy–Weinberg equilibrium (HWE) within sample sites were identified using the software package Arlequin (Excoffier, Laval, & Schneider, 2005). A total of 474 SNPs significantly deviating from HWE (*p *<* *.05 corrected to a false‐discovery rates (FDR) threshold of 0.02) across all six sites with *n* > 20 were removed from the library. SNPs were only removed if they were below MAF or HWE thresholds in all six of the larger sampling sites because wide divergences were expected between the distal populations in this study. Accordingly, rare SNPs were still retained if they were informative in at least one of the sites. Finally, SNPs associated with X‐ or Y‐linked chromosomes were screened among the 202 individuals with known sex using the full association test in Plink™ (Purcell et al., 2007), to ensure that only autosomal loci were retained in the dataset. The final SNP library contained 17,523 loci with an average call rate of 0.900 (*SE* ±0.001), average read depth of 15.994 (±0.059), and an average repeatability of 0.986 (±0.001). PAV markers for phylogenetic analyses were filtered manually to retain the most informative marker set across all individuals and taxa. PAV loci were removed based on a MAF of <2% among sample sites (*n* > 20) and technical reproducibility of less than 100%. A total of 22,387 PAV loci were retained for phylogenetic analysis across 250 individuals.

### 2.4 Assessing genetic diversity within sampling locations

To evaluate genetic diversity within and across sample sites, standard diversity indices including mean observed heterozygosity (*H*_o_), mean nonbiased expected heterozygosity (*H*_e_), and Wright's inbreeding coefficients (*F*_is_) were calculated through Genetix V4.05.2 (Belkhir, Borsa, Chikhi, Raufaste, & Bonhomme, 1996). Partial digestion during genotype by sequencing has previously been reported to result in null alleles, which can lead to inflated estimations of *F*_is_ (Andrews, Good, Miller, Luikart, & Hohenlohe, 2016; DaCosta & Sorenson, 2014). In order to address this issue, within‐site and locus‐by‐locus *F*_is_ estimates were calculated again with 1,000 permutations in Genetix V4.05.2 (Belkhir et al., 1996) using stringently filtered, site‐specific SNP libraries from which all loci were removed that did not robustly conform to HWE within the site being analyzed (*p *<* *.05 corrected to an FDR threshold of 0.20). These reduced datasets were more likely to omit informative or possible outlier loci, but minimized the likelihood of containing null alleles that could have affected accurate estimations of *F*_is_ within individual sites (DaCosta & Sorenson, 2014). All other within‐site diversity indices were consistent between the two filtering methods, but *F*_is_ was reported using both methods for comparison. As inbreeding affects the whole genome, a homogeneity test comparing all locus‐by‐locus *F*_is_ values was further conducted within each site to determine whether any positive observations of *F*_is_ were resulting from inbreeding behavior (as per Andrade, Medeiros, & Solferferini, 2005).

To assess individual genome‐wide diversity and inbreeding measures, standardized multilocus heterozygosity (sMLH) and internal relatedness (IR) were calculated for all individuals using the R package *Rhh* (Alho, VÄLIMÄKI, & MERILÄ, 2010). The 1 − proportion of shared alleles (*A*_S_) individual distance was calculated for each individual pair using the “propShared” command in *adegenet* (Jombart, 2008). The percentage of polymorphic loci (PPL), average individual multilocus heterozygosity (Av. MLH), proportion of rare alleles (*A*_R_; MAF < 0.05), and proportion of private alleles (*A*_P_) were calculated for each of the six sites (with *n* > 20) using custom scripts in Microsoft Excel™. To assess the effective population sizes (*N*_eLD_) and sibship structure at sampled locations, a subset of 500 loci was selected from the SNP database to be used in these analyses. These 500 loci were selected for having a minimum MAF of 0.05 within all sites and were then filtered for having the highest call rate, repeatability, and read depth among the remaining loci. Filtering SNPs for these analyses helped ensure many of the simplifying assumptions used in the calculations were met (see Do et al., 2014; Jones & Wang, 2010; Waples, 2006; Waples & Do, 2010). *N*_eLD_ was calculated with NeEstimator v2.0 (Do et al., 2014) using the linkage disequilibrium option. The proportions of full and half‐sibling pairs were calculated using the software program COLONY v2.0.6.1 (Jones & Wang, 2010).

### 2.5 Addressing broad‐scale divergence

Genetic differences among the six sites with sample sizes >20 were evaluated using Weir and Cockerham's unbiased *F*‐statistics (Weir & Cockerham, 1984) using Arlequin (Excoffier et al., 2005). The impact of geographic distance on genetic divergence (Mantel, 1967) was assessed by linearly regressing the pairwise *F*_st_ values between each site on their geographic distance using the software: GraphPad Prism™ (v6). Hierarchical analysis of molecular variance (AMOVA) among individuals and sample sites in different groupings was calculated in Arlequin (Excoffier et al., 2005). A discriminant analysis of principal components (DAPC) using the R package, *adegenet* (Jombart, 2008), was conducted for the genotypes of sampled animals obtained from the six sites where *n* > 20. An optimal A‐score test was run on this analysis using the same package, and the DAPC was run again using the optimal number of principal components and discriminant functions and visualized through a DAPC density plot. Individual genomic relationships among all samples were calculated and visualized using the NETVIEW (v0.5.1) pipeline (Steinig, Neuditschko, Khatkar, Raadsma, & Zenger, 2016) at *k*‐NN values between 10 and 60. Nei's standard genetic distances (Nei, 1978) and their significance were calculated among samples from the six larger sites (*n* > 20) with 1,000 permutations using Arlequin (Excoffier et al., 2005). The mean pairwise distances were then used for tree construction based on the neighbor‐joining (NJ) method in Mega6 (Tamura, Stecher, Peterson, Filipski, & Kumar, 2013). The resulting tree was then esthetically edited in FigTree (v1.4.2) to illustrate the inferred clustering relationships among the six primary sample sites (*n* > 20) in this study.

### 2.6 Identifying signatures of selection

Outlier analyses were used to identify candidate loci under directional selection among the six sites with *n* > 20, following both a frequency‐based approach in Lositan (Antao, Lopes, Lopes, Beja‐Pereira, & Luikart, 2008) and a Bayesian method in BayeScan (Foll, 2012). Both of these programs can run the risk of identifying false positives during outlier discovery (Narum, Buerkle, Davey, Miller, & Hohenlohe, 2013). To reduce this possibility, and putatively identify loci under directional selection, this study isolated overlapping outlier loci between these two programs (as per Jacobs et al., 2017). Samples from three ecologically and spatially separated sites within Western Australia (Fremantle, Mandurah, and Emu Point) and eastern Australia (Gulf St. Vincent, Port Phillip Bay, and Stanley) were compared within each region separately as the eastern and western sites were too divergent to be analyzed together (see Villemereuil, Frichot, Bazin, François, & Gaggiotti, 2014; Whitlock & Lotterhos, 2015). Directional outlier loci were selected for tree construction within the Western Australia region if both programs jointly identified them as directional outliers at FDR of 0.01. However, BayeScan, which is more robust to type I errors but can be more sensitive to high background *F*_st_ levels of the two packages (Lal et al., 2016; Narum & Hess, 2011), did not identify outlier loci within the eastern region at low FDR thresholds. Therefore, directional outlier loci were reported for this region and used in subsequent tree construction if they were identified by Lositan at an FDR or 0.01 and in BayeScan up to an FDR of 0.36.

The resulting directional outlier loci for both the western and eastern regions were used in tree construction by calculating the pairwise genetic distances (1 − proportion of shared alleles) using the “propShared” command in *adegenet* (Jombart, 2008). These pairwise values were then illustrated for both regions using the NJ tree method in Mega6 (Tamura et al., 2013). Due to the relaxed FDR used for identifying directional loci among the eastern sites in BayeScan, this NJ tree was used for explorative purposes only. Any interpretations of selection among the eastern sites derived from this analysis were made with extreme caution. A third NJ tree was also constructed, using the same methodology as above, but using all neutral loci for comparison. Finally, the sequences of all identified directional outlier loci were compared against the NCBI nucleotide database and the *Octopus bimaculoides* genome assembly (Albertin et al., 2015) for biologically relevant matches using Blast2Go™ software.

### 2.7 Phylogenetic reconstruction & evolutionary distances

Phylogenetic relationships among all individuals were reconstructed based on both the SNP and dominant loci (DArTseq PAVs) using maximum likelihood (ML) and Bayesian methods. For both analyses, data from the *H. fasciata* sister taxa were included as an out‐group. The ML analysis was conducted using the software RAxML v8.2 (Stamatakis, 2016) incorporating the ASC_GTRGAMMA[X] and ASC_BINCAT[X] site‐specific heterogeneity models for SNP and PAV loci, respectively (see Leaché, Banbury, Felsenstein, de Oca, & Stamatakis, 2015). For both ML analyses, the ascertainment bias correction (–asc‐corr) was set to “Lewis” and the rapid bootstrap algorithm with “autoMRE” (Pattengale, Alipour, Bininda‐Emonds, Moret, & Stamatakis, 2009) and best ML tree option selected (Stamatakis, 2008). In order to determine whether heterozygous site variation biased the phylogenetic reconstruction analysis, the SNP ML analysis was rerun using the repeated random haplotype sampling (RRHS) approach with 5,000 trees according to Lischer, Excoffier, and Heckel (2013). Bayesian inference of phylogenetic relationships used only the PAV dataset in MrBayes v3.2.6 package (Ronquist et al., 2012). In order to reach convergence, a subset of the 248 individuals that best reflected the PAV ML tree topology was used for Bayesian analysis. The analysis incorporated two runs of 100,000,000 generations, with each run comprising eight independent chains. A temperature of 0.10 was set for the heated chains, with a sampling frequency of 1,000 and burn‐in fraction of 25%. The Dirichlet prior for state frequencies was set at (40, 60), matching the frequencies of “0” and “1” PAV scores present in the dataset. Convergence was also independently assessed using Tracer v1.6 (Rambaut, Suchard, Xie, & Drummond, 2014). All resulting phylogenetic consensus trees were visualized and esthetically edited using the software FigTree v1.4.2 (http://www.molecularevolution.org/software/phylogenetics/figtree). In addition, the levels of phylogenetic distance among all pairs of individuals were calculated using the F84 evolutionary model for SNPs and the modified restriction method for PAVs (DNAdist and Restdist respective programs) in the Phyllip v3.695 analysis package (Felsenstein, 2005).

## 3 RESULTS

### 3.1 Genetic diversity

Among sample sites, mean observed heterozygosity (*H*_o_) ranged from 0.076 to 0.166, and mean nonbiased expected heterozygosity (*H*_e_) ranged from 0.086 to 0.250 (Table 1). All sample sites deviated significantly from Hardy–Weinberg equilibrium (*p *<* *.001). Wrights inbreeding coefficients (*F*_is_) ranged from 0.043 to 0.182 after rigorous filtering for null alleles (Table 1), but homogeneity tests of these coefficients across all loci revealed that locus‐by‐locus *F*_is_ was significantly heterogeneous within all sites (*p *=* *.000; Table S1). sMLH and IR ranged from 0.572 to 1.225, and 0.478 to 0.732, respectively (Table 1). Samples from the Stanley site returned the lowest values for *H*_o_, *H*_e_, *F*_is_, and sMLH, with the correspondingly highest results for IR and *A*_S_ (Table 1). The Fremantle and Mandurah sites returned the highest values of *H*_o_, *H*_e_, sMLH, PPL, and *A*_R_ while having the lowest proportions of half‐sibling pairs, *A*_S_ and IR values (Table 1). The Port Phillip Bay and Stanley sites had remarkably high proportions of half‐sibling pairs and the two highest scores for *A*_S_, despite having relatively low *F*_is_ scores (Table 1). The effective population sizes based on linkage disequilibrium (*N*_eLD_) ranged from 43.0 in the Gulf St. Vincent site to 1,794.1 in the Fremantle site (Table 1). However, the Mandurah and Port Phillip Bay sites returned *N*_eLD_ values of infinity. The *N*_eLD_ estimates should be regarded with some caution, as the assumption of random sampling may not have been met at all sites (Do et al., 2014; Waples & Do, 2010).

**Table 1:** Genetic diversity indices for the six *H. maculosa* sampling sites (*n* > 20) based on 17,523 SNP markers (“site filtered” *F*_is_ was calculated based on site‐specific subsets of loci stringently filtered for HWE; *N*_eLD_ and Prop

Site name	State	Location	*n*	*N*_eLD_ (95% CI at *p *=* *.05)	PPL	*H*_o_	*H*_e_	*F*_is_ (all loci; site filtered)	Av. MLH (±*SE*)	sMLH (±*SE*)	IR (±*SE*)	*A*_S_ (*SE* <0.001)	*A*_R_ (< 0.05 MAF)	*A*_P_	Prop. sibling pairs (full siblings; half siblings)
FRE	WA	Fremantle	91	1,794.1 (1,198.7–3,519.6)	0.908	0.166	0.250	0.339; 0.108	0.172 (±0.002)	1.225 (±0.015)	0.478 (±0.007)	0.787	0.170	0.053	0; 0.028
MAN	WA	Mandurah	37	Infinite	0.828	0.152	0.228	0.339; 0.182	0.158 (±0.002)	1.116 (±0.013)	0.472 (±0.006)	0.803	0.150	0.005	0; 0.024
ALB	WA	Emu Point, Albany	35	283.3 (231.9–362.6)	0.610	0.140	0.176	0.210; 0.095	0.146 (±0.004)	1.037 (±0.026)	0.490 (±0.012)	0.857	0.113	0.007	0.003; 0.210
SA	SA	Gulf St. Vincent	22	43.0 (40.2–46.3)	0.486	0.114	0.153	0.267; 0.175	0.119 (±0.002)	0.853 (±0.013)	0.594 (±0.006)	0.876	0.070	0.005	0.009; 0.238
VIC	VIC	Port Phillip Bay	22	Infinite	0.402	0.089	0.112	0.213; 0.153	0.093 (±0.003)	0.668 (±0.022)	0.686 (±0.01)	0.912	0.092	0.001	0; 0.784
TAS	TAS	Stanley	36	468.8 (347.7–713.4)	0.363	0.076	0.086	0.120; 0.043	0.080 (±0.002)	0.572 (±0.013)	0.732 (±0.006)	0.935	0.118	0.003	0.033; 0.684

Sibling Pairs were calculated based on a subset of 500 of the most informative loci). PPL stands for the percentage of polymorphic loci within each site. All *F*_is_ values were estimated from 1,000 permutations at *p *< .001. *A*_S_ stands for the proportion of shared alleles averaged among individuals for each site. *A*_R_ was calculated by the number of alleles having MAF less than or equal 0.05 among polymorphic loci within each site.

### 3.2 Broad‐scale divergence

**Figure 3:**
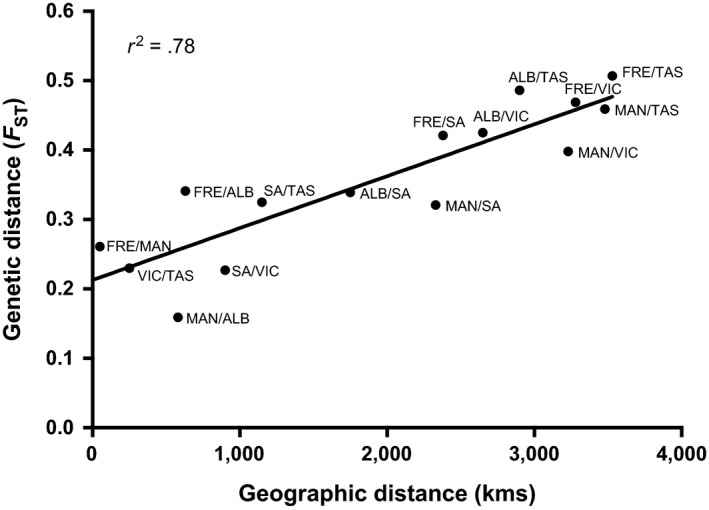
There was a significantly positive relationship between Wrights genetic distance (*F*_st_) and geographic distances (kms) between each sampling site. The solid line represents the linear regression: *y* = (7.477e−5)*x* + 0.213; *p *<* *.001

Pairwise *F*_st_ values based on Weir & Crockerham's unbiased distances are provided in Table 2 for the six sites with sample sizes >20. Values ranged from 0.159 between the Mandurah and Emu Point sites, which were located ~580 km apart, to 0.507 between the Fremantle and Stanley sites, which were the most geographically separated sites at ~3,530 km apart (Table 1). Genetic distances significantly increased with geographic distance (linear regression: *F*_1,13_ = 45.97, *p *<* *.001; Figure 3). The *r* ^2^ value of this regression revealed that geographic distance explained 78% of the variation in genetic differences; however, comparisons of the three west coast sites were exceptions to this pattern and the *F*_st_ value between the Mandurah and Emu Point sites fell well below the regression line (Figure 3).

**Table 2:** Pairwise *F*_st_ values are shown for each combination of sampled locations based on Weir & Crockerham's unbiased distances (Weir & Cockerham, 1984) with 1,000 permutations on the bottom left of the matrix

	FRE	MAN	ALB	SA	VIC	TAS
FRE	*	0.119	0.167	0.253	0.297	0.320
MAN	0.261	*	0.061	0.149	0.193	0.216
ALB	0.341	0.159	*	0.135	0.178	0.202
SA	0.421	0.321	0.339	*	0.056	0.082
VIC	0.469	0.398	0.425	0.227	*	0.041
TAS	0.507	0.459	0.486	0.325	0.230	*

Nei's standard genetic distances (Nei, 1978) based on 1,000 permutations are given in the top right side of the matrix. All *F*_st_ values have a significance of *p* < .001, and all Nei's standard genetic distances have a standard error of less than or equal to 0.003.

**Figure 4:**
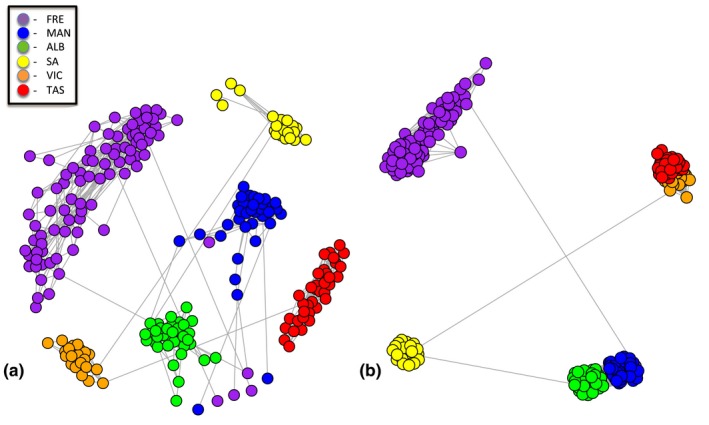
The genomic clustering of all sampled individuals using an isolation by state constructed using the NETVIEW V5.0 pipeline is visualized at (a) *k*‐NN = 15; and (b) *k*‐NN = 55

Both NETVIEW (*k*‐NN = 15) and DAPC analyses revealed that genotyped individuals primarily formed unique clusters based on broad geographic sampling location (Figures 4a and S1). However, two individuals sampled at the Fremantle site fell into the same cluster as other samples from the Mandurah site that was located approximately 50 km away from where they were obtained. Interestingly, NETVIEW analysis (Figure 4a) for Fremantle and Mandurah indicated substructuring and higher diversity within these sites, which was also supported by genetic diversity indices (Table 1). Based on AMOVA hierarchical analysis, the maximum amount of genetic variance was observed at the individual site level (47.22%; *p *<* *.001). The next highest level of variation was observed when the sampled sites were clustered into the four genetic groups: Fremantle; Mandurah and Emu Point; Gulf St. Vincent; Port Phillip Bay and Stanley. This arrangement accounted for the maximum amount of genetic variation among groupings (35.34%; *p *<* *.001), while 11.66% of variation was among sites within groups (*p *<* *.001) and 53.01% of variation among individuals within sites (*p *<* *.001). This grouping arrangement is inconsistent with both geographic distribution and the listed distributions of *H. maculosa* and WBRO (Norman, 2000), in that it suggests the individuals sampled from the Mandurah site are more genetically aligned with individuals from the Emu Point site than they are from the adjacent Fremantle site (Figure 2). The demarcation of these four genetic groups is further supported by the pairwise *F*_st_ data (Table 2), NETVIEW analysis at *k*‐NN = 55 (Figure 4b), and the NJ tree based on Nei's standard genetic distances (Figure S2).

### 3.3 Signatures of selection

A high proportion of directional outlier loci (*n* = 196, alpha range: 1.267–1.938) were jointly identified by both statistical methods at an FDR of 0.01 among the Western Australian sites (Fremantle, Mandurah, and Emu Point; Table S2). A total of 729 directional outlier loci were identified among the eastern Australian sites (Gulf St. Vincent, Port Phillip Bay, and Stanley) by Lositan at an FDR of 0.01. However, BayeScan analysis did not identify any outlier loci that overlapped with Lositan results at low FDR thresholds suggesting that outlier loci might be rarer among the eastern range of *H. maculosa*. At FDRs of 0.01 in Lositan and 0.36 in BayeScan, eleven overlapping directional outlier loci were cautiously identified (alpha range: 0.659–1.381; Table S2).

**Figure 5:**
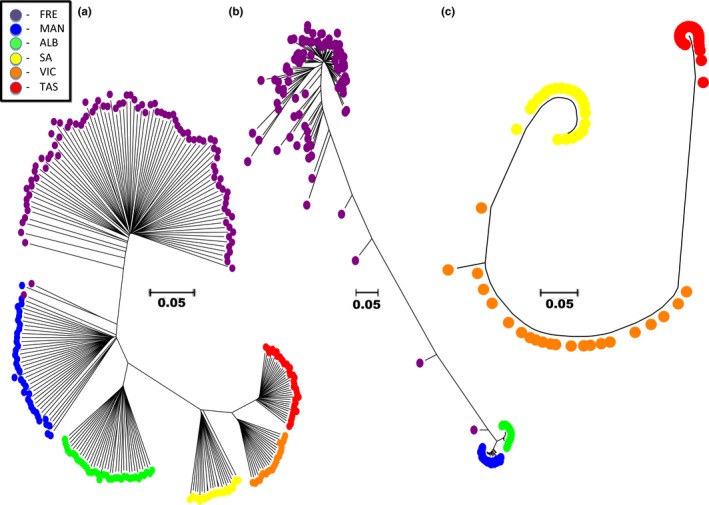
The relationships between individuals sampled from different locations are shown using the neighbor‐joining method based on pairwise “1 − proportion of shared alleles” among (a) the 17,316 neutral loci for all sampled individuals; (b) the 196 directional outlier loci jointly identified by Lositan and BayeScan analyses among the three west coast sites; and (c) the eleven candidate directional outlier loci identified by Lositan at an FDR of 0.01 and BayeScan at an FDR of 0.36. The legend at the top left of the figure displays the colors representing the site where individuals were sampled

When comparing the 17,316 neutral loci among individuals sampled from all six sites using pairwise values of “1‐proportion of shared alleles,” six clusters consistent with geographic proximities of sample sites were observed (Figure 5a), with branch lengths between sites consistent with *F*_st_ and Nei's genetic distances (Table 2). When using the same analysis for the 196 directional loci identified among the Fremantle, Mandurah, and Emu Point sites, the Mandurah and Emu Point sites clustered tightly together and were both separated from the Fremantle site via notably increased branch lengths (Figure 5b). Furthermore, the level of individual diversity among Mandurah and Emu Point was greatly reduced compared to Fremantle, which displayed larger and more variable, individual branch lengths. Interestingly, two individuals from the Fremantle sample site clustered with individuals from the Mandurah site in the neutral loci NJ tree (Figure 5a). However, both of these individuals migrated back toward the Fremantle cluster in the outlier NJ tree possibly reflecting partial adaptive variation in these individuals (Figure 5b).

Among the Gulf St. Vincent, Port Phillip Bay, and Stanley sites, the NJ tree based on the eleven candidate outlier loci revealed slightly longer branch lengths between all three sites compared to the neutral loci tree, but overall topology was similar (Figure 5c). Accordingly, local adaptation was present among the three eastern sites but less pronounced than in the western sites. When annotating outlier loci through Blast2Go™ software, no biologically meaningful matches were identified.

### 3.4 Phylogenetic reconstruction & evolutionary distances

**Figure 6:**
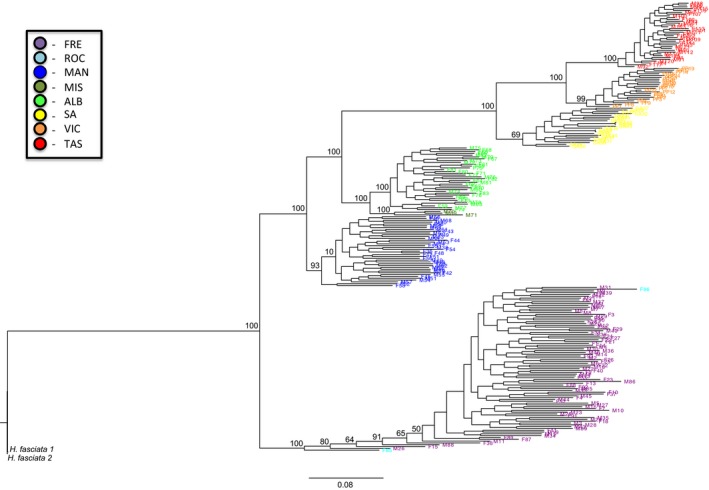
A ML tree for all 248 *H. maculosa* group samples from the eight sampling locations based on 100,000 bootstraps and 17,523 SNP loci. Two samples of the sister taxon *H. fasciata* are included as an out‐group. The bootstrap values are listed to the top left of major nodes. Sample names are color‐coded to their sampling location, as per the legend in the upper left, with the out‐group samples left in black

Based on both SNP and PAV phylogenetic inference methods, sampled individuals from the *H. maculosa* group formed distinct clades consistent with geographic proximities of individuals (Figures 6, S3 & S4). All clades were well supported in the ML and Bayesian analyses, providing strong confidence in the interpretation of phylogenetic reconstructions. There was no difference in tree topology or relative branch lengths when reconstructing trees using the RRHS ML methodology (Lischer et al., 2013), indicating that heterozygous sites were not biasing SNP ML tree reconstruction (data not shown). The placement of the *H. fasciata* out‐group clade shifted between SNP and PAV analyses (Figures 6 and S3). This might have been due to the large amount of divergence among all groups, as well as the potentially limited resolving power of branch lengths using PAV loci (see below). Among all analyses, the two individuals from Rockingham were well‐dispersed within the Fremantle clade, which suggests that these two sampling locations might be part of a larger single clade. The three samples from Misery Beach formed a separate clade basal to Emu Point indicating that these geographically close populations have most likely diverged in only relatively recent evolutionary history from one another. Individuals within the Fremantle clade displayed longer and more variable branch lengths compared to the other localities, which is consistent with higher diversity indices and a larger *N*_eLD_ observed in this site (Table 1). Upon comparison of relative branch lengths between all clades and the *H. fasciata* out‐group, large evolutionary divergence was apparent among all sampled sites for the *H. maculosa* group (Figures 6, S3 and S4). Consistent with the hypothesis of an existing sister taxon in Western Australia (Norman, 2000), there was as much genetic divergence between Fremantle and the three eastern *H. maculosa* sites (range: 0.279–0.332) as between *H. fasciata* and all sample sites for the *H. maculosa* group (range: 0.232–0.377) based on the SNP ML tree reconstruction and F84 genetic distances (Table 3). However, genetic divergences between Fremantle and the Mandurah and Emu Point sites, as well as between Mandurah and Emu Point compared to the eastern sampling sites, were substantially less than with the *H. fasciata* out‐group (Table 3) which suggests a clinal species pattern across this range. The relative divergence among sample sites and the *H. fasciata* out‐group was consistent in the PAV tree reconstructions (ML and Bayesian; Figures S3 and S4) and modified PAV genetic distance (Table 3). However, divergence estimated by the PAV markers was less pronounced overall. This reduction in relative branch length differences was primarily a function of the PAV loci and their loss of informative sites through the dominantly scored “0” or “1” classification (Lischer et al., 2013). Nonetheless, this constraint has not been shown to affect overall tree topology, particularly for closely related or recently diverged taxa (Althoff, Gitzendanner, & Segraves, 2007; Lischer et al., 2013).

**Table 3:** The F84 SNP genetic distances (below diagonal) and the modified PAV genetic distances (above diagonal) are given below between each of the sample sites with *n* > 20 and the sister taxon *H. fasciata*

	*H. fasciata*	FRE	MAN	ALB	SA	VIC	TAS
*H. fasciata*	*	*0.107*	*0.104*	*0.105*	*0.110*	*0.114*	*0.117*
FRE	*0.255*	*	0.053	0.057	0.068	0.074	0.076
MAN	*0.232*	0.176	*	0.042	0.053	0.058	0.060
ALB	*0.251*	0.198	0.094	*	0.048	0.052	0.054
SA	*0.321*	0.279	0.166	0.139	*	0.032	0.035
VIC	*0.359*	0.316	0.196	0.165	0.081	*	0.026
TAS	*0.377*	0.332	0.209	0.179	0.094	0.056	*

Genetic distances between *H. maculosa* (also WBRO) sampling sites and *H. fasciata* are given in italics. All F84 SNP genetic distances have a standard error less than or equal to 0.002. All modified PAV genetic distances have a standard error less than 0.001.

## 4 DISCUSSION

Genetic data presented here indicate that individuals sampled from the *H. maculosa* group follow a clinal species pattern across their geographic range, with the geographic extremities displaying levels of genetic divergence consistent with that of sister taxa. Furthermore, genetic divergence even among adjacent sampling sites in this study was remarkably high compared to studies of other cephalopods (Doubleday et al., 2009; Higgins et al., 2013; Keskin & Atar, 2011; Moreira, Tomás, & Hilsdorf, 2011; Pérez‐Losada et al., 2002; Reichow & Smith, 2001; Shaw et al., 1999; Zheng et al., 2009). These findings suggest that the high levels of observed genetic divergence among sampling sites are a result of this holobenthic species having insufficient gene flow among populations to counteract the strong effects of random drift, thereby creating a genetic IBD pattern along southwestern and southern coasts of the Australian continent. Additionally, differences in strong selective pressures between geographic locations, as detected by outlier analyses, are suggested to increase the genetic dissimilarities of geographically separate populations of the *H. maculosa* group. Together, these data reveal that life history traits and ecological factors are rapidly driving genetic divergence, and possibly speciation within this taxon.

Within sample sites, levels of both observed and expected heterozygosity were quite low compared to other genetic studies in cephalopods (Higgins et al., 2013; Kassahn et al., 2003; Moreira et al., 2011; Pérez‐Losada et al., 2002; Reichow & Smith, 2001; Shaw et al., 1999; Zheng et al., 2009). In part, this is due to differences in estimating heterozygosity between SNP and microsatellite markers, which were used in the above studies (see Vignal, Milan, SanCristobal, & Eggen, 2002), as well as the near impossibility of being able to eliminate all null alleles from the SNP library (Andrews et al., 2016; DaCosta & Sorenson, 2014). Nonetheless, the low levels of heterozygosity observed here might also reflect the limited dispersal and gene flow of this species group (Tranter & Augustine, 1973), leading to aggregations of highly related individuals. Heterozygosity scores were lowest for the Stanley site (*H*_o_ = 0.076; *H*_e_ = 0.086; Av. MLH = 0.08; sMLH = 0.572), along with the highest observed IR (0.732) and proportions of half siblings (0.684). These samples were obtained over a 1‐month period from a commercial fishery that only fished over a ~22 km^2^ area of relatively homogenous benthic habitat. However, the Stanley site also had the lowest *F*_is_ score (0.043 after within‐site HWE filtration) and second largest *N*_eLD_ estimate (468) observed among sample sites in this study. This, in combination with the observation that inbreeding coefficients were significantly heterogeneous at all sites, suggests that although highly related individuals are likely to occur within close proximity as they do in Stanley, genetic evidence infers that inbreeding might be extremely rare. Both *H*_o_ and *H*_e_ were highest at the Fremantle and Mandurah sites, where samples were obtained over ~61 and ~220 km^2^ areas, respectively, and these sites also yielded the two lowest levels of half‐sibling pairs and IR. The highest values for *F*_is_ were observed at the Mandurah site. However, due to the low levels of relatedness and large sampling area for this site, the higher *F*_is_ observed there was likely a result of Wahlund effect (Sinnock, 1975). The influence of Wahlund effect on *F*_is_ at the Mandurah site is further suggested by the substructuring patterns observed by Netview analysis for individuals from both the Mandurah and Fremantle (Figure 4a).

The juxtaposition of high levels of interrelatedness (and sibling pairs) with comparatively low, uncorrelated, and significantly heterogeneous inbreeding coefficients throughout the sites sampled in this study suggests that this species group might possess a mechanism for inbreeding avoidance. The low dispersal ability of this taxon, which results in the occurrence of closely related individuals within small areas, could leave populations of this species group particularly prone to inbreeding depression (Charlesworth & Charlesworth, 1987). Significantly positive inbreeding coefficients have been recorded previously in the golden cuttlefish, *Sepia esculenta* Hoyle, 1885 (Zheng et al., 2009), which also has a limited dispersal capacity. However, it is possible that the relatively lower *F*_is_ values observed in the current study could be due to the mating system of *H. maculosa* and/or their sister taxa. Hapalochlaena maculosa females are selective of their mates, males spend different amounts of time copulating with particular females, and both sexes copulate with multiple partners within their single breeding season (Morse et al., 2015). It is possible that members of the *H. maculosa* group can avoid inbreeding by either preferentially copulating with nonrelated partners (Pusey & Wolf, 1996), or by mating with several partners and allowing postcopulatory processes to bias fertilization to compatible gametes (Tregenza & Wedell, 2000; Zeh & Zeh, 1997). This latter possibility might also help to explain the extreme prevalence of polyandry in both this species group (Morse et al., 2015; Tranter & Augustine, 1973) and possibly the holobenthic cephalopods in general (Hanlon & Messenger, 1998). Further studies investigating the paternity patterns among gentotyped candidate parents with known relatedness would be necessary to verify this hypothesis.

Where estimated, effective population sizes were highly variable among sample sites (Table 1). The relatively larger population estimate at Fremantle suggests that this species can be common in some areas and that individuals might aggregate together due to habitat selection and/or breeding areas to better facilitate its synchronous terminal‐breeding season (Tranter & Augustine, 1973). Due to the cryptic nature of the *H. maculosa* group, aggregation behavior has not been documented in the wild. However, seasonal aggregations to facilitate breeding behavior have been suggested by observations of predictable abundance and patterns of size structuring in the Cockburn Sound, WA, in addition to synchronous egg‐laying events observed in laboratory settings (P. Morse personal observations). It is unknown why Gulf St. Vincent had a lower *N*_eLD_ compared to other sample sites, but it is possible the limited sample size and observed sampling of related individuals over a smaller area might impacted this calculation.

The observed *F*_st_ values among sample sites were very high compared to all comparable studies of population divergence in cephalopods (Doubleday et al., 2009; Higgins et al., 2013; Keskin & Atar, 2011; Moreira et al., 2011; Pérez‐Losada et al., 2002; Reichow & Smith, 2001; Shaw et al., 1999; Zheng et al., 2009). Additionally, *F*_st_ values increased proportionally with geographic distance, implicating an IBD pattern for gene flow, consistent with *O. pallidus* (Higgins et al., 2013), several species of cuttlefish (Kassahn et al., 2003; Pérez‐Losada et al., 2002), and many terrestrial animals (Wright, 1943). This pattern strongly indicates that populations of the *H. maculosa* group are finely structured over distance due to their lack of a planktonic dispersal phase. Such a scenario suggests that the genetic connectivity of this species group might be highly susceptible to geographic barriers such as benthic topography or degradation of suitable habitat (Slatkin, 1973). However, pairwise genetic differences closely fit their expected values predicted by geographic distance, so no obvious genetic bottlenecks or specific barriers to gene flow were identified among sample sites in this study.

The only exceptions to this pattern were that the *F*_st_ value between Mandurah and Emu Point sites was much lower than expected based on geographic distance, whereas the *F*_st_ value between Fremantle and Mandurah was slightly higher. Interestingly, samples from Fremantle and Emu Point were both obtained in relatively shallow water (4–10 m depth), whereas samples from the Mandurah site were obtained from greater depths (17–28 m). It is possible that the deeper habitats around the Mandurah, WA, act as a barrier to dispersal and gene flow between the Fremantle and Mandurah sites, delineating the genetic groups between these two sites. Results from the AMOVA, DAPC, and NETVIEW analyses all indicated that limited gene flow is present between adjacent sample sites, however support the above results in that animals sampled from the Mandurah site share more genetic similarities with the Emu Point site (*F*_st_ = 0.159, and ~580 km away) than individuals in the adjacent Fremantle site (*F*_st_ = 0.261, and only ~50 km away; Figures 3, 4 and S1). A morphological survey of the ecotypes occurring over this range would be helpful by determining which of these ecotypes might or might not have a functional ink sac. The above genetic data suggest that the delineation between *H. maculosa* and the WBRO might be further north on the western coast than previously reported (Norman, 2000).

The evolutionary divergence of individuals among sites sampled in this study was further supported by phylogenetic analyses using the sister taxon *H. fasciata*. Consistent with the previous separation of the WBRO from *H. maculosa* (Norman, 2000), phylogenetic reconstructions in this study indicated that the ecotype sampled from Fremantle is more genetically distant from *H. maculosa* ecotypes sampled from eastern sites than it is from the described sister taxon *H. fasciata* (Table 3; Figures 6, S3 and S4). Additionally, genetic divergence was sufficiently strong among all six of the primary sample sites in this study to justify investigation into the presence of potentially cryptic subspecies occurring at some or all of these sites. However, these data also indicate that gene flow occurs across the entire sampled region of this study through occasional migrations between adjacent populations. This suggests that the *H. maculosa* species group is in fact a species gradient that follows a clinal pattern across the proposed *H. maculosa* and WBRO distributions. It is recommended that future studies address morphological variation of this group, in order to complement the genetic data provided here and help in further defining the delineations between ecotypes within this potential species complex (e.g., Meudt, Lockhart, & Bryant, 2009).

On examination of the 207 directional outlier loci within the *H. maculosa* group genome, it was evident that there were distinct signatures of selection present among the different sites. Although local adaptation was indicated in each of the six larger sites, the greatest divergence in selective pressures was observed between individuals from Fremantle and individuals from both the Mandurah and Emu Point sites. Furthermore, it was suggested that individuals from the Mandurah and Emu Point sites might be under similar selective pressure and/or have possibly been separated from the Fremantle site due to a recent genetic bottleneck or range expansion. This pattern adds further support to the delineation between the Fremantle and Mandurah genetic groups, and suggests either environmental pressures (Mayr, 1963) or selective breeding behaviors (Wright, 1940) might be acting to reinforce the divergence of the Mandurah and Emu Point individuals from the Fremantle ecotype. It is noteworthy that the two Fremantle individuals, who had previously clustered with Mandurah within the DAPC, NETVIEW, and neutral loci figures, began to recluster toward the Fremantle group in the west coast outlier tree. This supports that some migration does occur between the Fremantle and Mandurah sites and that these two individuals, who were obtained near Fremantle, might have been descendants from recent migrants coming from Mandurah.

Selective pressures were subtler among the eastern sample sites (Gulf St. Vincent, Port Phillip Bay, and Stanley). No outlier loci were identified using BayeScan analysis for the eastern sites at low FDR thresholds, which was possibly due to less pronounced local adaptation in this region and also the sensitivity of BayeScan to large differences in background *F*_st_ among sites (Lal et al., 2016; Narum & Hess, 2011). However, upon examination of the eleven overlapping outlier loci identified at a more relaxed FDR, samples from the eastern sites did show a slight increase in branch lengths when compared to neutral loci (Figure 5). This suggests that different selective pressures might be present among these three sites, although their impact on the *H. maculosa* genome is less defined within this region. None of the outlier loci identified in this study matched any biologically meaningful genes during blast analyses. This is most likely due to the general paucity of genomic sequencing studies in octopods and the lack of annotation within octopod genomes (c.f. Albertin et al., 2015; Ogura, Ikeo, & Gojobori, 2004). The increasing availability of genetic markers and techniques may enable future studies to easily link loci under directional selection to biologically meaningful regions of cephalopod genomes.

Together, these findings reveal strong divergence among populations of the *H. maculosa* species along its range, most likely due to the limited dispersal capacity associated with this taxon's holobenthic and brief 7‐month life history (Tranter & Augustine, 1973). These genetic differences are sufficient to justify the categorization of two distinct sister taxa and/or investigation into the possibility of several cryptic subspecies. However, these data also indicate that taxonomic delineations within this group should be made with caution, as gene flow occurs across the species range through allele sharing between adjacent populations. It is hoped that future annotations of the entire *H. maculosa* genome might enable the identification of what types of directional selection are occurring along the species range, and the role that local adaptation might play in possible speciation within this group. Parallel studies addressing the phylogeny of distinct genetic groups within the greater blue‐ringed octopus (*H. lunulata*) might be useful to compare findings in this study to a tropical congeneric possessing a planktonic larval stage (Overath & Boletzky, 1974). Finally, it is also indicated that fine‐scale genomic studies in the *H. maculosa* group are warranted. The general processes shaping the broad‐scale genomic structure of this species along its geographic range have been identified here. However, there is still much to be learned from this enigmatic taxon via investigating patterns of relatedness, possible subspecies delineations, and sex‐biased dispersal at much finer geographic scale (100s–10s km).

## 5 CONCLUSIONS

This study provides the first molecular investigation within the *Hapalochlaena* genus and the first genetic assessment of a holobenthic cephalopod across its entire range. These findings strongly indicate that *H. maculosa* and the WBRO form a single clinal species, following a genetic IBD pattern, common to terrestrial animal taxa (Wright, 1943) and other marine organisms that lack a planktonic life history phase (Barbosa, Klanten, Puritz, Toonen, & Byrne, 2013; Higgins et al., 2013; Kassahn et al., 2003; Pérez‐Losada et al., 2002). There was evidence of strong genetic divergence among sampling sites along the *H. maculosa* group distribution, most likely due to the limited dispersal capacity and short 2‐month life cycle of the species. Phylogenetic reconstructions including the *H. fasciata* sister taxon further support that the divergence between *H. maculosa* ecotypes at both ends of their distribution exceeds that observed between some heterospecifics in this genus. However, no two adjacently sampled locations showed comparable divergence to the *H. fasciata* out‐group. Therefore, the taxonomic identities and geographic ranges of *H. maculosa* and WBRO require revaluation. Parallel studies with additional sister taxa (e.g., *Hapalochlaena lunulata*) will be useful as a comparison of habitats and life histories, in addition to providing phylogenetic context for the genomic divergence observed here between holobenthic members of the *Hapalochlaena* genus. Further molecular studies, investigating relatedness and sex‐biased dispersal of *H. maculosa* at a more localized scale, will be useful for additional insights into the behavior of this cryptic taxon.

## CONFLICT OF INTEREST

None declared.

## AUTHOR CONTRIBUTIONS

All authors contributed to revisions of the text and the final content of the manuscript. P. Morse and K. Zenger contributed to the experimental design. P. Morse and J. Finn collected genetic samples. M. Meekan provided a research vehicle for fieldwork. S. Kjeldsen and P. Morse performed DNA extractions and analyzed data under guidance from K. Zenger. P. Morse and K. Zenger organized funding for this research. P. Morse was the primary author.
