# Supplementary figures and images for: Genome‐wide comparisons reveal a clinal species pattern within a holobenthic octopod—the Australian Southern blue‐ringed octopus, Hapalochlaena maculosa (Cephalopoda: Octopodidae)
Source: Ecol Evol. 2018 Jan 25;8(4):2253–67. doi: 10.1002/ece3.3845 (PMC5817145; doi:10.1002/ece3.3845)

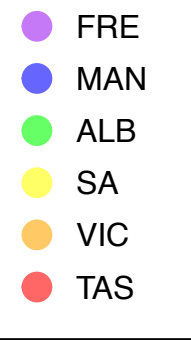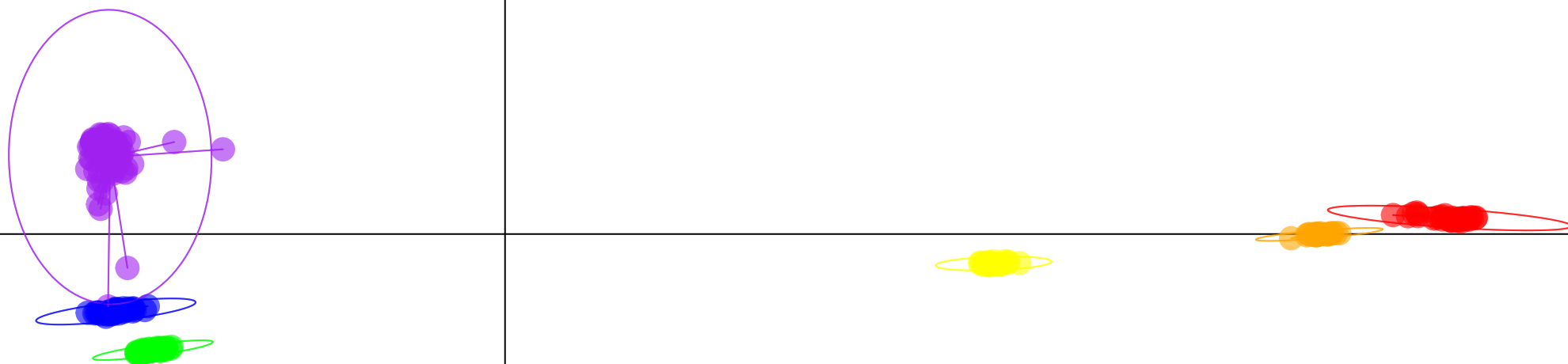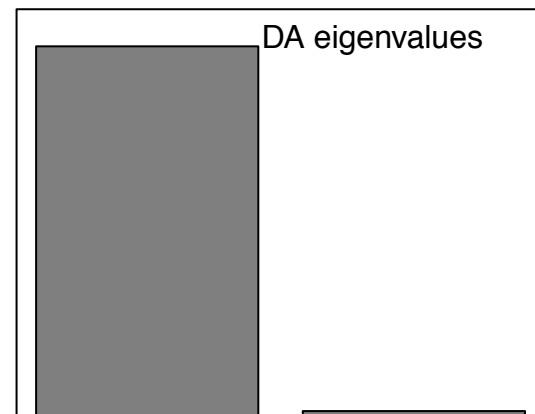

Supplement: Supplementary file 1 [file ECE3-8-2253-s001.pdf]

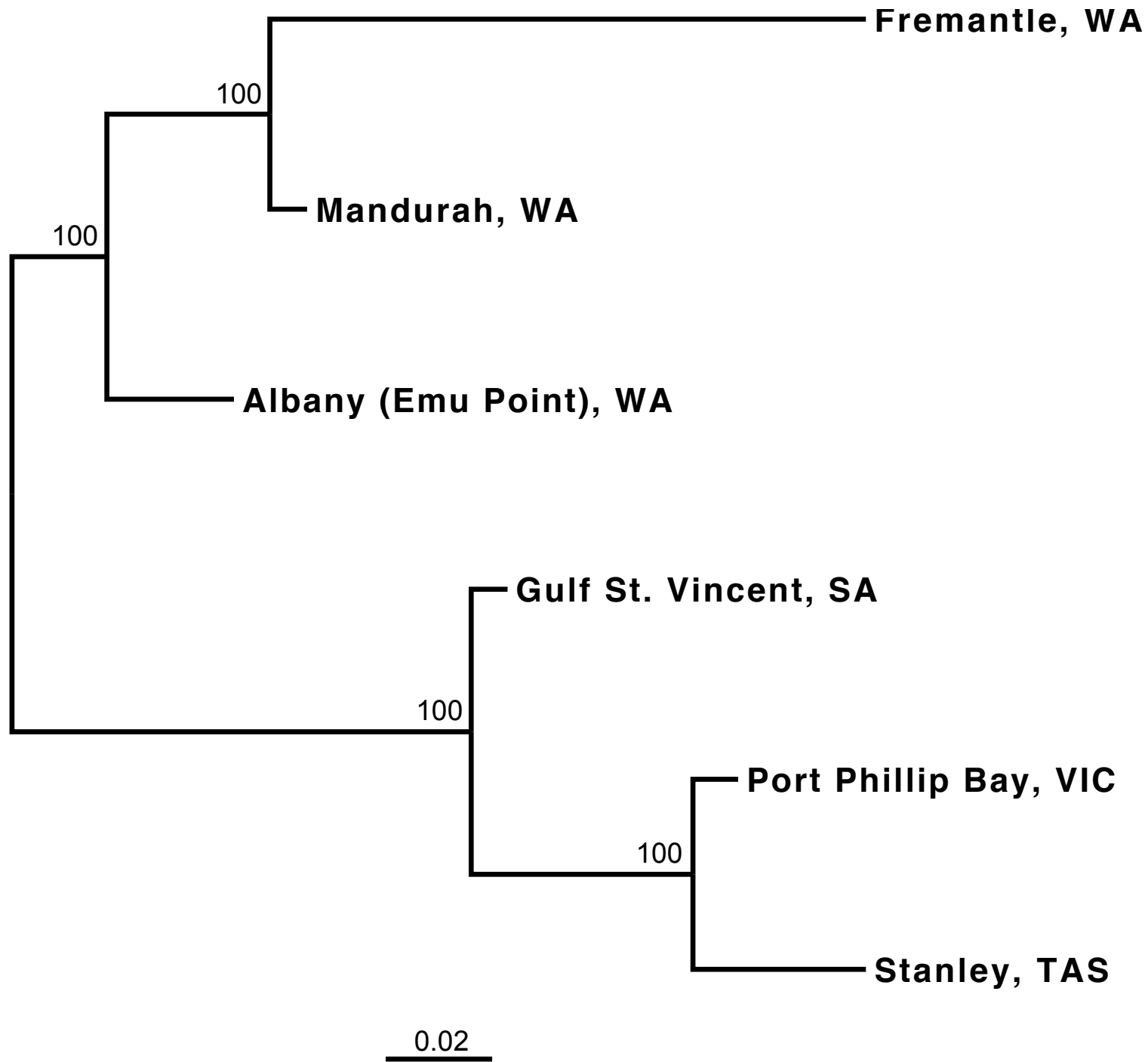

Supplement: Supplementary file 2 [file ECE3-8-2253-s002.pdf]

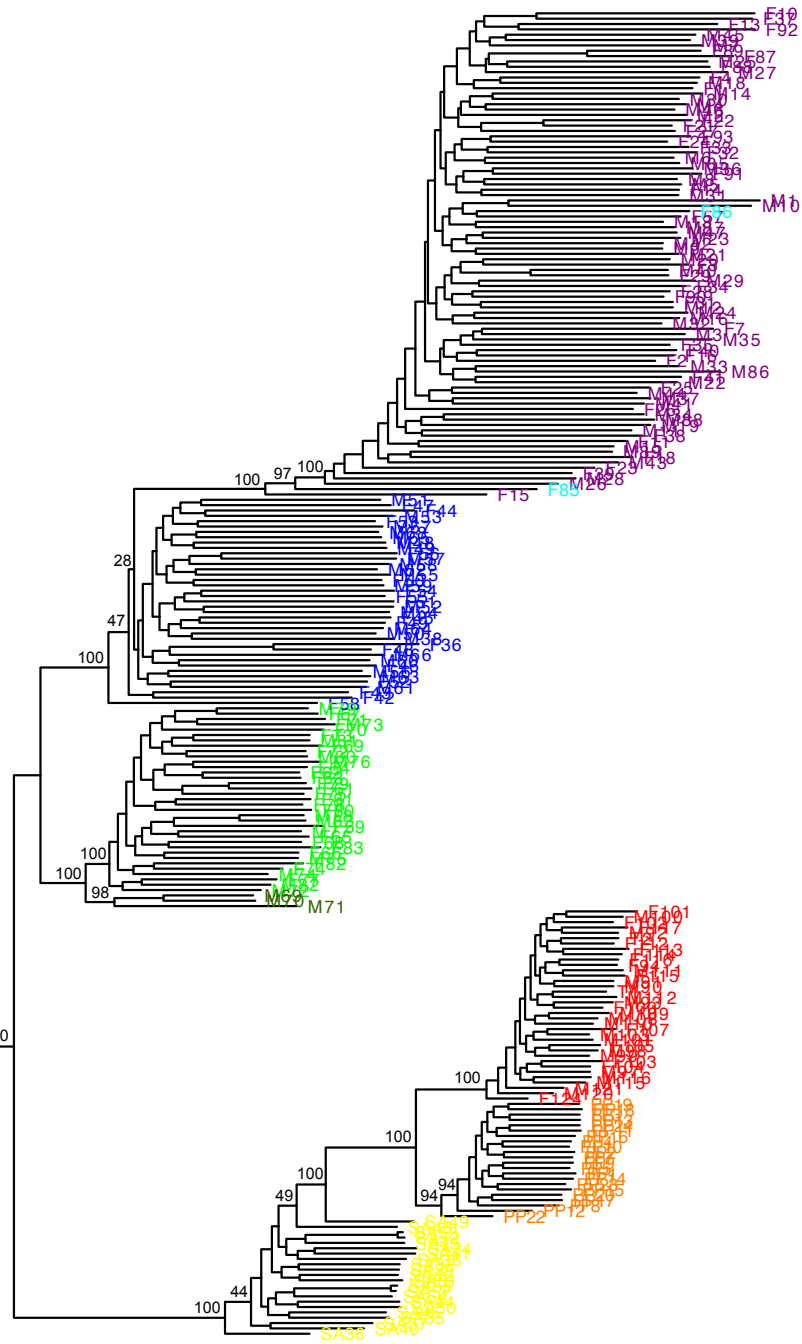

0.3

Supplement: Supplementary file 3 [file ECE3-8-2253-s003.pdf]

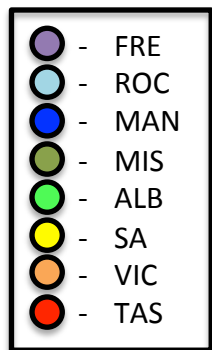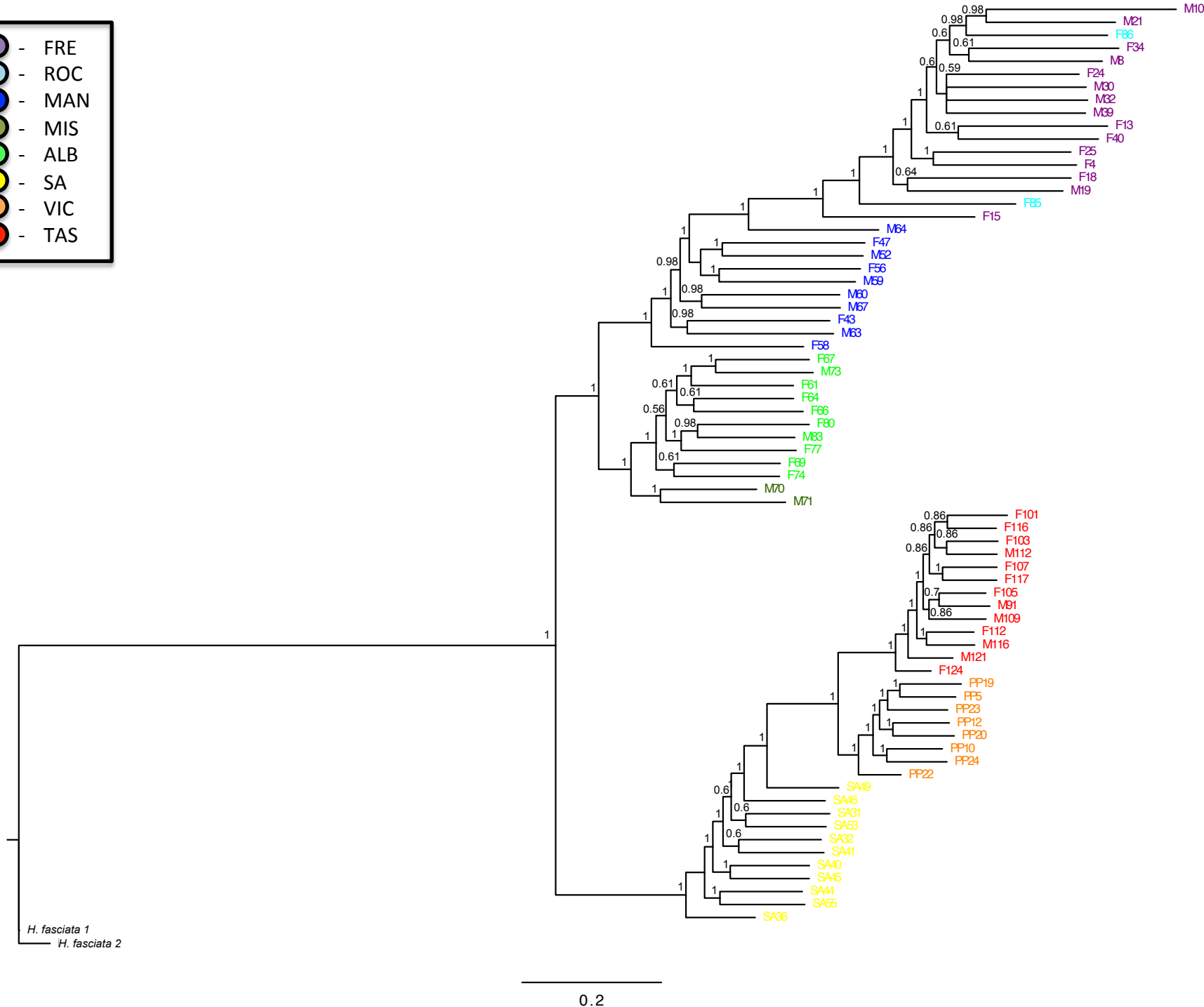

Supplement: Supplementary file 4 [file ECE3-8-2253-s004.pdf]
