# Supplementary material for: Genome‐wide comparisons reveal a clinal species pattern within a holobenthic octopod—the Australian Southern blue‐ringed octopus, Hapalochlaena maculosa (Cephalopoda: Octopodidae)
Source: Ecol Evol. 2018 Jan 25;8(4):2253–67. doi: 10.1002/ece3.3845 (PMC5817145; doi:10.1002/ece3.3845)
Supplement: Supplementary file 5 [file ECE3-8-2253-s005.docx]

**Supplementary Table 1.** The results for the homogeneity tests of inbreeding coefficients (*F_is_*) within each site (n > 20) are given below. Tests were run on subsets of loci that were stringently tested for HWE within each population to exclude the possibility of null alleles. *F_is_* estimates were significantly heterogeneous among loci at all sites.

| **Site** | **Χ^2^** | **d.f.** | ***p*** |
| --- | --- | --- | --- |
| FRE | 19,514.324 | 7,783 | 0.000 |
| MAN | 18,444.332 | 9,043 | 0.000 |
| ALB | 15,915.317 | 7,366 | 0.000 |
| SA | 10,340.797 | 5,897 | 0.000 |
| VIC | 127,737.807 | 5,332 | 0.000 |
| TAS | 7,873.197 | 4,574 | 0.000 |
