# Supplementary material for: Genome‐wide comparisons reveal a clinal species pattern within a holobenthic octopod—the Australian Southern blue‐ringed octopus, Hapalochlaena maculosa (Cephalopoda: Octopodidae)
Source: Ecol Evol. 2018 Jan 25;8(4):2253–67. doi: 10.1002/ece3.3845 (PMC5817145; doi:10.1002/ece3.3845)
Supplement: Supplementary file 6 [file ECE3-8-2253-s006.docx]

**Supplementary Table 2.** The numbers of candidate outlier loci discovered by Lositan and BayeScan analyses are shown below. Overlapping directional outliers from both analyses at FDR = 0.01 were used for tree construction of the western sites in Figure 5. No overlapping outlier loci were detected by BayeScan among the eastern sites at low FDR thresholds. Therefore, directional loci identified by Lositan among the eastern sites at an FDR of 0.01 were used in tree construction if they were jointly identified by BayeScan with an FDR up to 0.36. These outliers among the eastern populations need to be interpreted with caution, however the high Fst values and alpha scores of these loci strongly suggest that they occur at diversifying regions of the genome for individuals sampled from these locations.

| **Compared Sites** | **Lositan Outliers**  **(FDR = 0.01)** | | **BayeScan**  **Directional Outliers** | **Overlapping Directional Outliers used in Analyses** | **Average Fst of**  **Overlapping Outliers**  **(± S.E.)** | | **Average BayeScan Alpha Score of Overlapping Outliers**  **(± S.E.)** |
| --- | --- | --- | --- | --- | --- | --- | --- |
|  | **Stabilising** | **Directional** |  |  | **Lositan** | **BayeScan** |  |
| FRE x MAN x ALB | 2,065 | 1,181 | 540 (FDR = 0.01) | 196 | 0.896 (± 0.003) | 0.577 (± 0.002) | 1.561 (± 0.009) |
| SA x VIC x TAS | 422 | 729 | 12 (FDR = 0.36) | 11 | 1.000 (± 0.000) | 0.682 (± 0.018) | 1.043 (± 0.092) |
