# Supplementary material for: Genome‐wide comparisons reveal a clinal species pattern within a holobenthic octopod—the Australian Southern blue‐ringed octopus, Hapalochlaena maculosa (Cephalopoda: Octopodidae)
Source: Ecol Evol. 2018 Jan 25;8(4):2253–67. doi: 10.1002/ece3.3845 (PMC5817145; doi:10.1002/ece3.3845)
Supplement: Supplementary file 7 [file ECE3-8-2253-s007.docx]

**Supporting information**

**Figure S1** A DAPC scatter plot, created with the R package *adgenet*, displays the extent of structuring between each sample site with n > 20 based on Discriminant Functions 1 and 2. The applicable A-score analysis revealed that all meaningful structuring between sites was explained by these two discriminant functions.

**Figure S2** The evolutionary relationships between sampled locations with n > 20 are illustrated using the Neighbour-Joining reconstruction method with Nei’s standard genetic distances averaged over 1,000 permutations. The optimal tree is shown with the sum of branch lengths = 0.367 and bootstrap values to the left of each node.

**Figure S3** A maximum-likelihood tree the 248 *H. maculosa* group samples from the eight sample sites used in this study based on 100,000 bootstraps with DArTseq PAV dominant markers. Two samples of the sister taxon *H. fasciata* are included as an out-group. The bootstrap values are listed to the top left of major nodes. Sample names are colour-coded to their sample site, as per the legend in the upper left, with the out-group samples left in black.

**Figure S4** A Bayesian reconstruction of a 74-sample subset of the *H. maculosa* group used in this study and two *H. fasciata* sister taxon samples based on PAV dominant markers. The posterior probabilities of each divergence are listed next to each node. Sample names are colour-coded to their sample site, as per the legend in the upper left, with the out-group samples left in black.
